# A Complicated Case of Labour

**Published:** 1910-07-02

**Authors:** Henry Robinson


### GYNÆCOLOGY.

### A COMPLICATED CASE OF LABOUR.

By HENRY ROBINSON, M.A., M.D.(Cantab.).

In putting on record the following case of obstetrical complications I am fully aware that, although several of the points in connection with it are unusual, there are probably few practitioners who could not narrate equally interesting and difficult cases from their own experience. It is the combination of several obstetrical and other difficulties that makes the history worth recital.

The patient is a Frenchwoman, about 24 years of age. She has been married nearly two years, and has been pregnant but once, the occasion of the events now to be narrated. At about three o'clock one morning, being then, according to her calculation, about 27 weeks (just over six calendar months) pregnant, she awoke with abdominal pain and a rather free hæmorrhage from the vagina. No doctor or nurse had been engaged, and her husband called me in forthwith. I found the patient in bed, with a trickle of blood oozing slowly from the vagina, and I was shown a chamber containing clotted blood to the extent, roughly estimated, of eight ounces. She appeared in excellent condition, with normal temperature, and a good pulse of 80 to the minute.

On abdominal examination it was found that the uterus resembled in size that of a seven or even seven and a half months' pregnancy: it was contracting intermittently, but quite painlessly. I ought to add that the initial pain when the hæmorrhage first began was referred to both sides of the abdomen, to the hypogastrium, and to the sacral region. No fœtal parts were clearly made out, except a foot near the upper pole and on the right-hand side. At this stage I debated whether the signs were best explained by an error of calculation as to the date of conception, by assuming that menstruation had occurred once after fertilisation, or by some such complication as accidental hæmorrhage or excess of liquor amnii.

On vaginal examination the cervix was found not only non-dilated, but not even taken up: this must be a most unusual state of affairs, particularly in view of the typical labour pain which had awakened the patient. In front of the cervix a fœtal head was felt presenting; in the posterior fornix nothing but a boggy soft mass could be palpated. A diagnosis of placenta prævia was made, and a decision arrived at to empty the uterus without delay. The patient had not only no nurse but no attendant of any kind, nor any preparations of any sort for maternity. I contented myself, therefore, with applying a gauze pad to the vulva. After breakfast, having secured the services of a capable nurse, I found the patient practically *in statu quo*: hæmorrhage had all but ceased, and was, in fact, no greater than in normal menstruation. I anæsthetised lightly with chloroform, and packed the vagina, after douching, quite full of iodoform gauze previously boiled. Five rolls were introduced.

In the afternoon I returned and found the patient with regular and fairly good labour pains. A little blood had escaped externally, but the plugs had been packed so tight that they contained only a very little more when removed. The cervix was dilated sufficiently to admit the tip of the forefinger, and the edge of the placenta, which had just covered the internal os, was easily felt. I then secured the help of a brother practitioner to administer an anæsthetic, and without any marked difficulty gradually dilated the cervix manually until it admitted three fingers. The presenting fœtus was then turned by bipolar version, the membranes were ruptured, and a foot brought down. The hæmorrhage during these manœuvres was not excessive; but deeming the fœtus to have in any case but a poor chance, I brought down the other foot and speedily delivered a male infant of 2 lb. weight, which at once began to breathe. At the same time I realised that another fœtus was still inside the uterus, and on vaginal examination I felt it presenting by the vertex, and almost through the os uteri. This one, therefore, I did not turn: it was born naturally, but dead; it was of $1\frac{3}{4}$ lb. weight and the male sex. The unusual size of the uterus was thus, of course, amply explained.

The third stage of labour was tedious, and a good deal of hæmorrhage occurred. After about fifty minutes, however, a single very large and intact placenta, with the membranes, left the uterus and was easily expressed from the vagina. The uterus retracted well, and never gave any trouble at all from the conclusion of the third stage onwards. The total loss during the whole labour I estimate at 30 ounces, possibly rather more; the patient was distinctly blanched by the time everything was concluded (some 16 hours after the first onset of symptoms), and her pulse-rate was 104.

It was only in the puerperium that the discovery was made of a double mitral lesion, well compensated, however; and inquiry then elicited a history of two attacks of acute rheumatism. The puerperium was complicated by a severe vaginal tear in the upper part, not involving the perineum. This may possibly have been due to the distension caused by the packing. The split did not extend through into the rectum, but presumably almost did so, as it became the seat of a septic infection which extended into the broad ligament but did not affect the uterus: this delayed convalescence considerably. The first child died two days after its birth.

The unusual points about the case may be briefly summarised thus: The association of placenta prævia with twins; the onset of symptoms as early as the twenty-seventh week of pregnancy; the association of hæmorrhage and pains with a cervix not at all obliterated; the delivery of a living child at this early period of gestation, especially in a twin pregnancy and with the placental condition; the addition of valvular disease of the heart to the other complications; and the unusual vaginal injury to which reference has been made.

